# Coping Strategies for Pertussis Resurgence

**DOI:** 10.3390/vaccines11050889

**Published:** 2023-04-24

**Authors:** Xuanxuan Nian, Hongbo Liu, Mengyao Cai, Kai Duan, Xiaoming Yang

**Affiliations:** 1 National Engineering Technology Research Center for Combined Vaccines, Wuhan 430207, China; 2 Wuhan Institute of Biological Products Co., Ltd., Wuhan 430207, China; 3 China National Biotech Group Company Limited, Bejing 100029, China

**Keywords:** pertussis resurgence, vaccines, DTaP, DTwP

## Abstract

Pertussis (whooping cough) is a respiratory disease caused primarily by *Bordetella pertussis*, a Gram-negative bacteria. Pertussis is a relatively contagious infectious disease in people of all ages, mainly affecting newborns and infants under 2 months of age. Pertussis is undergoing a resurgence despite decades of high rates of vaccination. To better cope with the challenge of pertussis resurgence, we evaluated its possible causes and potential countermeasures in the narrative review. Expanded vaccination coverage, optimized vaccination strategies, and the development of a new pertussis vaccine may contribute to the control of pertussis.

## 1. Introduction

Pertussis (whooping cough) is called ‘cough for 100 days’ in folk medicine and is an acute respiratory infectious disease [1]. Typical symptoms are a series of short paroxysmal coughs and high-pitched “whooping” sounds. In the 1920s, scientists found that *Bordetella pertussis* (*B. pertussis*) was the most important pathogen that caused pertussis. It is a small, non-motile, strictly aerobic, Gram-negative bacillus that colonizes the respiratory tract of humans, causing respiratory disease. In the 1950s, Pearl Kendrick, Grace Eldering, and Loney Gordon developed an efficient whole-cell Pertussis (wP) that was used in a Diphtheria, Tetanus, and whole-cell Pertussis (DTwP) combined vaccine. The emergence of DTwP and its sequential use in some developed countries have substantially reduced the incidence of pertussis [2]. However, wP prepared directly from *B. pertussis* is prone to adverse reactions. Therefore, vaccination coverage rates fell in many countries in the 1970s [3,4]. Scientific studies demonstrated that the combined vaccine for diphtheria, tetanus, and acellular pertussis (DTaP), prepared against the major toxins and antigens of *B. pertussis*, prevented pertussis with an efficiency similar to that of DTwP. Following successful clinical trials in the 1990s, the wP in many countries has been replaced by the safer acellular pertussis (aP) for pertussis vaccination [5,6,7,8]. The purified antigens of *B. pertussis* in aP include fimbrial proteins 2 and 3 (Fim2/3), filamentous hemagglutinin (FHA), pertussis toxin (PT), and pertactin (PRN) [9]. Fim2/3 is a pathogenic agent of *B. pertussis* and is expressed on the bacterial surface to promote bacterial binding to host cells [10]. FHA is involved in bacterial adhesion to ciliated epithelium [11]. FHA residues 1877–2250 have been found to elicit the most potent immune response and therefore constitute the most important immunogenic site [12]. PT is the main virulence factor of pertussis, which enters mammalian cells and affects immune cell function. For instance, PT inhibits the chemotaxis of phagocytes so that the phagocytes cannot reach the site of inflammation [13,14]. The World Health Organization “WHO position papers on Pertussis (2015)” recommended continuing with the use of wP and that countries that switched to aP should ensure additional booster doses and additional strategies such as maternal immunization; meanwhile, only aP-containing vaccines should be used for persons aged ≥7 years [15]. The global coverage of DTP (both DTaP and DTwP) ranges from 86–90% of the first DTP-containing vaccine and 81–86% of 3rd dose in 2008–2021. Compared to the global coverage of DTP in 2012–2019, the global coverage of DTP decreased in 2020–2022; the main reason may be because of the COVID-19 pandemic [16]. Still, the global incidence of pertussis keeps increasing year by year [6]. This phenomenon has aroused global concern, and the “pertussis resurgence” phenomenon has been successively reported not only in developed countries but also in many developing countries [4]. Relevant research data reported 24 million cases of pertussis and 160,000 deaths among children under 5 years of age worldwide in 2014. Most of the infections and deaths are associated with pertussis, especially in children in Africa and some developed countries [17]. Understanding the reasons for the resurgence of pertussis, optimizing existing vaccination regimens, and developing novel effective pertussis vaccines could be effective strategies to cope with the resurgence of pertussis.

## 2. Causes for Pertussis Resurgence

Pertussis resurgence might be attributed to the lower efficacy of the DTaP compared to the DTwP [18]. Serum antibodies induced by DTaP rapidly declined after the third and fourth dose of DTaP vaccinations without long-term protection [19,20]. Natural infection and immunization with wP were associated with the induction of strong T-helper 1 (Th1) and Th17 cell response, which promoted rapid antibody responses and cell responses after DTwP vaccination [21,22,23]. On the other hand, aP vaccines induce higher Th2, and Tdap booster vaccination in children induces lower vaccine antigen-specific humoral immunity and Th1 cell responses in aP-primed compared with wP-primed [24,25]. wP vaccination was significantly more effective than aP vaccination in conferring persistent protective immunity against *B. pertussis* due to the respiratory tissue-resident memory T cells (TRM cells) induced by wP [26]. Thus, DTaP-induced effective immune response to *B. pertussis* was lower than that induced by natural infection and DTwP vaccination. The Pertussis resurgence is also associated with the low mucosal immune response induced by aP and the coverage of the vaccine. A study assessing pertussis vaccination and anti-pertussis herd immunity levels in the target vaccination population in 2019 found that low percentages of vaccination coverage and low anti-pertussis herd immunity levels in the target vaccination population were two of the factors explaining pertussis resurgence and persistence worldwide [27]. In 2019, the mean prevalence of individuals with vaccine-induced pertussis immunity in the target pertussis vaccination population was 81.1% among the countries worldwide, ranging from ranging from 76.7% in the African region to 93.7% in the European region [27]. Anti-pertussis herd immunity could not be established worldwide in the target vaccination population against pertussis agents with Ro ≥ 10 because the required critical prevalence of 90% was too high to block pertussis transmission in the community [27]. Vaccine hesitancy also contributes to low vaccine coverage. Pertussis resurgence is also attributed to the variations in pertussis pathogens and epidemiological changes under vaccine selection pressure [28,29]. Currently, aP is being used in more than 100 countries and regions worldwide, and *B. pertussis* presents antigenic drift under immune selection pressure after vaccination. The ongoing genetic shift and gene loss of *B. pertussis* affected the protection efficacies of the existing vaccines [30], such as the discovery of pertactin-deficient clinical isolates and the emergence of the novel Ptx allele strain in the Ptx promoter region of pertussis toxin [31,32]. The prevalence of clinical isolates of B. pertussis deficient in PRN expression was almost exclusively described in countries that use aP vaccines [33,34,35,36]. These variants were more toxic and infectious, with greater epidemic selection advantages [37,38]. With the decline in immunity over time and lack of booster vaccination, older people became more susceptible to *B. pertussis* [39,40]. In particular, the presentation of pertussis was generally less severe in adolescents and adults, which often caused medical professionals to miss out on their diagnosis, making them a potential source of transmission [41]. Public awareness of this disease has been increasing, with the detection methods. The polymerase chain reaction (PCR) method can sensitively and rapidly identify *B. pertussis* in nasopharyngeal specimens and thus help improve the diagnostic level [42]. Traditional vaccination policy cannot control “pertussis resurgence” well. Therefore, new targeted prevention and control measures need to be taken based on the altered epidemic characteristics and transmission patterns of pertussis.

## 3. Optimize Vaccination Policies to Address Pertussis Resurgence

The vaccination policies should be optimized by establishing pertussis monitoring protocols, strengthening the pertussis monitoring, improving the detection level, understanding the actual incidence of pertussis, and then establishing prevention and control strategies according to its incidence and epidemic characteristics. Prenatal vaccination of pregnant women in the third trimester of pregnancy allows pregnant women to produce high levels of antibodies before delivery. These antibodies are transmitted to the fetus through the placenta. Then, infants are born with a high antibody titer to defend against pertussis infection [43]. DTaP vaccination in pregnant women has been advocated in more than 40 countries [44]. Among them, Canada advocated vaccination in the third trimester (between 27 and 32 weeks of gestation); the USA had a long window for vaccination (between 27 and 36 weeks of gestation); and England implemented a relatively early vaccination strategy (between 20 and 32 weeks of gestation) [45,46,47]. Despite the variation in the time of vaccination during pregnancy across different countries, all pregnancy vaccination strategies have been shown to significantly reduce *B. pertussis* infection in infants and young children, preventing approximately 70–90% of potential cases of pertussis disease and up to 90.5% of pertussis disease-related hospitalizations among infants under 3 months old [44,48]. In 2011, the Advisory Committee on Immunization Practices (ACIP) recommended that pregnant women and persons having close contact with an infant (aged 0–12 months) be vaccinated with Tdap [49]. Benefit-cost studies on booster immunization with increasing doses also supported the routine increase in pertussis vaccination in adolescents. Their impact on the most vulnerable group, infants under 6 months, is lower [50]. Booster immunization with pertussis at intervals of 10 years has shown good immunogenicity and safety in adolescence in clinical practice, while the impact of the booster immunization in adolescence on the most vulnerable group, infants under 6 months, is lower [51,52]. Many developed countries have adjusted their immunization strategies for pertussis, emphasizing the vaccination of pregnant women, adolescents, and adults (Table 1). Furthermore, Austria, Belgium, France, Germany, Greece, Italy, Luxembourg, and other countries have advocated that adults should be vaccinated every decade [53]. Around 50–55%, 6–8%, and ≤20% of infants and young children reportedly obtain an infection from their parents, grandparents, and siblings, respectively. Vaccination of the family members and individuals in close contact with them, ensuring strict protection of infants and establishing a “cocoon strategy”, could effectively reduce infection and mortality among infants and young children and was demonstrated to have cost-benefit [54]. According to WHO, the overall impact and cost-effectiveness of high vaccination coverage for the cocoon strategy was lower than maternal immunization which requires only one dose [55]. Because of the overall local coverage rate, some studies in the USA and Canada showed that the cocoon strategy did not confer many benefits, which might be attributed to the overall local coverage rate of the DTaP [56,57]. Therefore, many factors need to be considered before the implementation of the cocoon strategy.

## 4. Critical Needs for New Pertussis Vaccines

Optimizing immunization policies, which only reduce pertussis incidence by increasing vaccine coverage, cannot significantly enhance the efficacy and duration of immune protection provided by the vaccine. Therefore, novel pertussis vaccines are urgently needed to address this challenge. Innate immune mechanisms by dendritic cells, macrophages, neutrophils, natural killer cells, and antimicrobial peptides contribute to the control of pertussis infection. However, complete bacterial clearance requires cellular-mediated immunity mediated by Th1 and Th17 cells [58]. IFN-γ secreted by Th1 cells activates macrophages, promotes the production of opsonizing antibodies, and helps clear *B. pertussis* from the respiratory tract [59]. Th17 cells have also been evidenced to defend against *B. pertussis* infection by activating neutrophils [60]. The weak immune response induced by aP is partly due to the absence of Th17 induction, whereas Th2 did not play a significant role in response to *B. pertussis* infection [60]. The types of antigenic components in the existing aP are finite (two components: PT and FHA; three components: PT, FHA, and PRN; and five components: PT, FHA, PRN, and Fim2/3), resulting in a narrow spectrum of specific immune responses against pertussis strains and restricted protection against pertussis. In a clinical trial comparing 2-component aP, 5-component aP, and wP, only 5-component aP was found to achieve acceptable efficacy—75.4% for pertussis cases with paroxysmal cough for ≥21 days and 61.8% for all laboratory-confirmed pertussis cases, indicating that the addition of Fim antigens improved the efficacy of aP [61]. Therefore, the development of novel pertussis vaccines should focus on the increase in response spectrum and Th1, Th17, and TRM cells mediated responses. At present, the virulence and candidate antigens optimization of existing pertussis vaccine, the optimization of antigen design and adjuvant selection, development of live attenuated vaccines, and application of novel nucleic acid vaccines may be the current and future direction of vaccine development.

### 4.1. Virulence and Antigen Optimization of Existing Pertussis Vaccines

The use of the previously used pertussis vaccine, wP, has been almost eliminated because of more adverse reactions, including endotoxins such as lipo-oligosaccharide (LOS), which, although contributing considerably to the reactogenicity and efficacy of pertussis vaccines, are highly inflammatory. Chemical elimination of LOS significantly reduced the wP-induced endotoxin content (by 20%) and endotoxin-related toxicity (by up to 97%) without compromising the integrity, potency (antibody and T-cell response), and stability of wP [62]. The SA subunit of PT is the most immunogenic molecule because it contains a major protective epitope and has ADP-ribosyltransferase activity [63]. Genetically detoxifying the SA subunit of PT of *B. pertussis* using genetic engineering (by mutating the 9th residue arginine of the subunit to lysine and the 129th residue glutamic acid to glycine) to prepare pertussis strains with enzymatically inactive SA subunit reduced the complexity of traditional chemical detoxification processes and helped achieve more sustained immune protection [64].

Currently, the safety of aP is satisfactory, but the spectrum, depth, and persistence of aP-induced immune protection are poor. The addition of more antigenic components can effectively increase the immune protection provided by the existing aP. Adenylate cyclase toxin (ACT, CyaA) is the main virulence factor of *B. pertussis*, which is required for infection and is an effective immunogen and protective antigen. The addition of the C terminal toxin repeat domain (RTX) antigen of ACT to aP at the appropriate dose enhanced the protection of immunized mice against the *B. pertussis* challenge [65,66]. Adenosine cyclase toxin lacking adenylyl cyclase activity (ACT^Δ^), in combination with antigenic components of the pertussis vaccine, enhanced serum IgG antibody responses, induced NO and IFN-γ production in peritoneal macrophages and spleen cells. The increased immune response provided protection against the B. pertussis challenge [67]. AfuA (BP1605) is an antigen located on the bacterial surface. It is conserved in clinical isolates and expressed during *B. pertussis* infection, may be involved in iron acquisition during host colonization, and is a highly antigenic protein. In a previous study, immunization of mice with recombinant AfuA-induced opsonophagocytic antibodies was found to protect the mice against pertussis [68]. IRP1–3 is a dimeric membrane protein involved in iron uptake. IRP1–3 is conservatively expressed in clinical isolates of *B. pertussis* and positively regulated by iron starvation. Immunization of mice with recombinant IRP1–3 produced a strong antibody response that recognized native proteins on the bacterial surface, thus promoting effective phagocytosis of bacteria by human polymorphonuclear neutrophils (PMNs), which is a key protective approach against this pathogen. Thus, IRP1–3 exhibited a protective effect against *B. pertussis* infection in the mouse model [69]. The addition of rAfuA and rIRP1–3 protein to three commercial aP significantly improved the vaccine’s protective activity [68]. Ferromodulin (FeSOD) is an immunogenic protein that is a part of the surface immunoproteomes of *B. pertussis* I and Saadet strains [70]. Intraperitoneal injection of a combination of bacterial FeSOD protein and lipid monophosphate A (MPLA) to immunized mice induced strong IgG1, IgG2a, and IFN-γ responses and reduced bacterial colonization in the lungs of the injected mice [71]. Bordetella resistance to killing A (BrkA) is a virulence factor activated by phosphorylated BvGA. A triple pertussis DTaP vaccine, comprising PT, FHA, and recombinant BrkA protein (rBrkA), has been found to have comparable efficacy with commercial inanrixtrade vaccine in protecting the immunized mice against infections with pertussis Tohama I and 18-323 strain [72].

Bacterial biofilm (BF) refers to a large number of bacterial aggregation membrane-like substances wrapping themselves. It is formed by bacteria adhering to the contact surface, secreting polysaccharide matrix, fibrin, lipid proteins, etc. The formation of biofilms might enhance the virulence of *B. pertussis* in the human nasopharynx, and the antigens expressed at this stage may be potential targets for vaccination [73]. In a previous study, 11 protein antigens were obtained from the biofilms of *B. pertussis*. Of these, the content of surface-exposed bordetella intermediate protein A (BipA) was the most abundant, surface-exposed protein. A mice model proved that antibodies to BipA were found to efficiently opsonize bacteria [74]. In another study, upregulated expression of outer membrane protein assembly factor (BamB) was detected in the biofilms of PRN-deficient clinical isolate ID20 containing the ptxP3 allele, and lipopolysaccharide assembly protein L (ptD) was detected in the biofilms of the Tohama I and the ID20 strains; the abundant presence of these two proteins was also found in the biofilms of *B. pertussis* [75]. Extracellular DNA is also one of the main components of *B. pertussis* biofilms. The antibodies induced by *B. pertussis* biofilms can effectively block the transmission and respiratory infection of *B. pertussis*. The immunization of mice with a combination of rBamB-rLptD and DTaP in the biofilm components induced IFN-γ and IL-17a locally in the spleen and lymph nodes. It provoked IgG2a antibody responses, exhibited better protection efficacy against *B. pertussis* than DTaP alone, and was well tolerated [76]. DTaP has shortcomings in effectively preventing the recipient against clinical strains. However, the combination of BamB and LptD with DTaP can enhance the protective effect of DTaP on the clinical strains of pertussis and is clinically a very valuable vaccine candidate. These newly emerging antigenic components, if used effectively and reasonably in existing aP, will significantly improve the immunogenicity of these vaccines.

### 4.2. Enhancing the Degree of Immune Response with Outer Membrane Vesicular Vaccines

Outer membrane vesicles (OMVs) are secreted by bacteria into the external environment and are composed of several substances, such as outer membrane proteins, lipopolysaccharides (LPSs), and nucleic acids. OMVs have been used in two meningitis vaccines, which demonstrated the feasibility of OMVs as components of bacterial vaccines [77]. A total of 43 proteins of pertussis OMVs have been identified, such as PT, PRN, ACT-Hly, LOS, and other outer membrane or periplasmic proteins. Of these, 10 are associated with localization to the outer membrane or cytoplasm/membrane (containing signal peptides), 3 with periplasm localization, 1 with cytoplasmic membrane/periplasm localization, and 18 with cytoplasmic localization, and the remaining 11 proteins are of unknown origin [78]. Pertussis OMV vaccines contain a wide range of bacterial antigens in natural structures, which can induce a broader immune response without the risk of the excessive inflammatory response as observed in wP [78,79,80]. LOS in OMVs can cause some side reactions. However, the LOS endotoxin can be removed by the PagL enzyme. In 2011, Asensio et al. demonstrated, in a mouse model, that the OMVsBpPagL vaccine induced lower endotoxin levels than wild-type OMVs while its protective efficacy was as high as that of wP [81]. Similar to the results obtained with wP, TRM T cells were found in lung tissue after the vaccination of mice with pertussis-OMV vaccines. Additionally, these *B. pertussis*-specific splenic Th1 and Th17 memory responses, as well as CD4^+^ TRM cells producing IL-17 and IFN-γ in the lungs induced by pertussis-OMV, play an important role in maintaining immune persistence [82]. Parenteral injection of pertussis OMV vaccines has been shown to produce mixed Th1/Th2/Th17 and CD4^+^ TRM cell responses [83]. Vaccination of mice with pertussis OMVs combined with diphtheria and tetanus toxoids could induce long-term immune responses that persist for up to 8 months while effectively preventing challenges against a variety of pertussis mutants as well as PRN-negative isolates. The formulation of the pertussis OMV vaccine contains a large number of immunogens that could help avoid selection pressure generated by a single or a few protective vaccine antigens [84,85].

### 4.3. Exploration of Novel Adjuvants in the Use of Pertussis Vaccines

Lipopolysaccharide (LPS), a bacterial cell wall component of the wP, activates innate immune cells through toll-like receptor 4 (TLR4) and has good immunoreactivity [86]. As a purified component pertussis vaccine, aP lacks the natural pertussis-related component present in wP and therefore has a weaker immune activation ability. Licensed aP contains aluminum adjuvants that can induce Th2 and Th17 immune responses and stimulate humoral immune responses. However, the existing immunity may not be enough to prevent *B. pertussis* infection because of the lack of Th1 cellular immunity [60]. Some novel adjuvants can activate the functioning of innate immune cells, such as dendritic cells (DCs). These cells, in turn, produce adaptive immune responses to antigenic components in the vaccine, which increases the breadth of the immune response and improves the depth of the immune response, which is important for further optimization of the current early-life vaccination strategy [87]. Only a few classes of adjuvants are currently approved for use in vaccines for humans, but no relevant clinical outcomes have been reported for the novel adjuvanted pertussis vaccines. TLRs are transmembrane receptors predominantly expressed by innate immune cells. They can be divided into cell surface (TLR1, TLR2, TLR4, TLR5, and TLR6) and intracellular TLRs (TLR3, TLR7, TLR8, and TLR9). TLR agonists can associate innate immune responses with adaptive immune responses and may therefore be potentially used to enhance and accelerate the early immunity of aP vaccines [88]. Compared to traditional aluminum adjuvant, lipoprotein BP1569, which has a TLR2 agonist effect, activated DCs and macrophages and mononuclear cells, enhanced Th1 and Th17 cell responses and IgG2a antibody responses when combined with aP, and exhibited better protective effect against *B. pertussis* infection than equivalent aP prepared with aluminum adjuvant [89]. LP-GMP adjuvant, a combined adjuvant of a stimulator of interferon genes receptor (STING) agonist (c-di-GMP) and a TLR2 agonist from *B. pertussis* (LP1569), synergistically induced the production of IFN-β, IL-12, and IL-23, as well as the maturation of DCs. Parenteral immunization of mice with LP-GMP combined with aP promoted Th1 and Th17 responses and protected the mice against *B. pertussis* lung infection. Moreover, intranasal immunization induced *B. pertussis*-specific Th17 responses and respiratory CD4^+^ TRM cell responses secreting IL–17 and provided a high degree of protection against intranasal colonization and lung infection for at least 10 months [90]. The aP prepared with PT, PRN, and Fim2/3, in combination with a tri-adjuvant system of innate defense regulatory peptide IDR 1002, TLR3 agonist poly (I:C), and polyphosphazene, was prepared with LT-A/LTA (antigens externally associated with L-TriAdj/antigens internally associated with L-TriAdj) for aP cationic lipid nanoparticle vaccines. The animal model found that aP cationic lipid nanoparticle vaccines induced systemic and mucosal immune responses (Th1-type responses, IgG2a, and IgA antibody responses) after intranasal delivery in immunized mice, showed promising results, and could be used as a potential formulation for intranasal administration of pertussis vaccines [91]. The small molecule imidazoline TLR7/8 agonists adjuvant formulation impacted the delivery and immunocompetence of leukocytes, including DCs. Licensed DTaP, in combination with TLR7/8 agonists adjuvant, provoked Th1/Th17 T cells and IgG2c humoral responses after immunization in neonatal mice, which, in turn, overcame neonatal hyporesponsiveness to aP vaccine [92]. The aP formulated using novel adjuvants of aluminum hydroxide-adsorbed TLR 7 agonists enhanced *B. pertussis*-specific Th1 and Th17 responses and serum IgG2a/b antibodies and enhanced the protective efficacy of the aP against pertussis aerosol challenge [93]. Similarly, TLR7 agonist adjuvant encapsulated in lactide-co-glycolide (PLG) nanoparticles, combined with DTaP, significantly enhanced IgG and IgG2a antibody responses. However, the immunity enhancement was insignificant when the TLR7 agonist adjuvant was not encapsulated in PLG nanoparticles [94]. Adsorption of PT, PRN, FHA, and Fim by aluminum hydroxide with TLR 4 or 9 agonists resulted in aP-specific IgG antibody responses, better activated previous pertussis immune memory in mice, and enhanced protection through early bacterial control and accelerated clearance [95]. CpG adjuvant, as a widely studied TLR adjuvant, combined with aP alone induces weak IgG1 and IgG2 responses, but CpG combined with aluminum adjuvant or other components with adjuvant effect might induce stronger cellular and humoral immunity and provide protection against challenges with pertussis strains [96,97,98,99]. Currently, phase I clinical trials on the CpG-adjuvanted pertussis–diphtheria–tetanus combination vaccine have been completed in Australia. The results described above indicated that TLR agonists alone as an adjuvant for pertussis vaccines did not significantly enhance the immune effect of pertussis, and the combination use of TLR agonist and aluminum adjuvant could exert a greater adjuvant effect in pertussis vaccine.

In addition to TLR agonist adjuvants, other novel adjuvants have also been investigated in pertussis vaccines. Layered dihydroxide (LDH) is an efficient adjuvant platform that has been demonstrated to induce effective and persistent immune responses. Mg/Al-LDH, as a nanoadjuvant, significantly improved pertussis humoral immune responses [100]. Poly [di (sodium carboxylatophenoxy) phosphazene] (PCPP) can be used as a novel mucosal adjuvant for aP. It has been shown to enhance the responses of antigen-specific IgG and IgA in mice serum and bronchoalveolar lavage fluid and increase the number of IgG- and IgA-forming cells in the nasal passages, lungs, and submandibular glands. Further, it was also found to enhance Th1 and Th2 responses (i.e., high levels of IgG2a and IgG1 as well as IFN-γand IL-4) in mice models. aP combined with PCPP exhibited a strong protective effect against respiratory tract infections with *B. pertussis* [101].

### 4.4. Live Attenuated Vaccines May Be Dark Horses to Control Pertussis Resurgence

In order to induce protective systemic and mucosal immunity, vaccine candidates for live attenuated vaccines have been developed, including attenuated vaccine BPZE1, which has been assessed in several clinical trials [102,103,104]. It was shown in phase 2b trail that BPZE1 induced broad and consistent *B. pertussis*-specific mucosal secretory IgA responses, whereas the Tdap group in this trail did not induce consistent mucosal secretory IgA responses. BPZE1 was well tolerated, with mild reactogenicity and no serious adverse events related to the vaccination [105]. GamLPV, another Live attenuated vaccine, has already undergone phase I/Ⅱ clinical trials (ClinicalTrials.gov Identifier, NCT03137927, NCT04036526). Both the preclinical and clinical study of GamLPV provided the better immunogenicity and protective activity [106]. BPZE1 was prepared by the knockout of the *B. pertussis* gene that encodes for skin necrosis toxin and replacement of the A mpG gene of pertussis by the A mpG gene of *Escherichia coli*, thus greatly reducing tracheal cytotoxin production. Studies have shown that the BPZE1 vaccine induced CD4^+^ CD69^+^ CD103^+^ TRM cells in the nasal mucosa of mice. These cells, in turn, produced high levels of IL-17 and considerable levels of IFN-γ. IL-17-dependent and SIgA-mediated protect the nasal and lung tissues of the mice from *B. pertussis* infection [107,108]. BPZE1 stimulates DCs to secrete pro-inflammatory cytokines and regulatory cytokines to drive the polarization of Th1- and Th17-type cells and increase adaptive immune responses [109]. Ciaran M. Skerry et al. showed that high levels of pertussis-specific IFN-γ and low levels of IL-17, effective memory B cells, and IgG2a-predominant specific antibody responses in the mice also could be detected in mice one year after vaccination with the BPZE1 candidate vaccine [110]. These persistent specific IFN-γ and IL-17 cells and memory B cell responses effectively alleviated the pertussis resurgence [111]. TLR4-deficient mice with MyD88 knocked out were used to demonstrate that the early protection of BPZE1 might be mediated via TLR4 signal path, inducing an inflammatory response in the early stage and increased pertussis-specific antibody (and possibly T cells) responses in the later stage, all of which contributed to the clearance of *B. pertussis* [112]. At the same time, the transient colonization of the nasopharynx by attenuated strains induced IgG and IgA responses to pertussis-specific antigens [113].

### 4.5. Pertussis Nucleic Acid Vaccine

Despite limited studies on nucleic acid vaccines for pertussis, some promising experimental results remain. Immunization of mice with plasmid DNA expressing the SA subunit of PT (PTS1) (pcDNA/S1) of *B. pertussis* induced the production of anti-PT IgG antibodies. pcDNA/S1 showed significant protection against intracerebral challenge with lethal doses of *B. pertussis* [114]. Other studies have shown that although no PTS1-specific IgG response was detected in mice serum by intramuscular immunization of mice with a DNA vaccine encoding PTS1, the high levels of IFN-γ and IL-2 responses induced promoted the rapid clearance of *B. pertussis* infection in the mice lungs [115]. DNA vaccine encoding the toxic N-terminal 180-amino acid fragment of pertussis PTS1 (C180) induced the strongest protective immunity against PT after immunization of mice. A new pertussis DNA vaccine with low toxicity was prepared by modifying C180 by gene editing, which induced anti-PT IgG antibodies [116]. Further, DNA vaccines were constructed by combining the PTS1, PRN, and FHA genes, encoding the major antigens of pertussis. These vaccines induced the expression of specific antibodies IL-10 and IFN-γ in immunized mice and also protected mice from death due to intracerebral challenge from lethal doses of *B. pertussis* [117]. The further constructed recombinant pertussis DNA vaccines containing the PTS1, PRN, and FHA gene fragments and PRN gene fragments and plasmids expressing GM-CSF were used simultaneously to immunize mice and boost immunization with the antigen components of aP, which stimulated higher levels of antibodies [118].

## 5. Feasibility of Future Development of Pertussis Vaccines

Factors contributing to pertussis resurgence include the low immune potency of existing aP adjuvants, the regression of immunity in previously vaccinated individuals, the emergence of non-vaccine-type strains, and the mutation of new strains allowing them to escape the selection pressure of vaccines [119,120]. Increasing the number of immunizations and the vaccination regimen for pregnant women are only expedient measures at present. Therefore, the effectiveness of the vaccine must be improved, and new pertussis vaccines must be developed to prevent and control the infection caused by new mutant strains. However, most new promising pertussis vaccine candidates are in the preclinical stage. Their protective efficacy has only been assessed on animal models. There is no detailed supporting safety data, so the efficacy and safety of clinical trials will be a major challenge for their successful marketing [121]. In addition to the traditional antigens, PT, FHA, PRN, and Fim2/3, the new antigens explored (such as BF, BipA, ACT^Δ^, etc.) as well as OMV pertussis vaccines have the potential to be used in combination with tetanus and diphtheria vaccines, turning them into the novel pertussis–diphtheria–tetanus combined vaccines [65,67,73,81]. The combined vaccines prepared by recombinant pertussis antigen and diphtheria and tetanus components demonstrated the feasibility of optimizing the components of pertussis vaccines in the combined vaccine [122,123].

The safety and social benefits of pertussis vaccines with new adjuvants for children and adults require careful consideration. Introducing new adjuvants to aP vaccines requires consideration of both the protective efficacy of the vaccines and extensive preclinical and clinical research. The impact of the new adjuvant on the immunogenicity of co-administered antigens such as diphtheria and tetanus components in the short or long term is still uncertain. Studies on TLR agonist-adjuvanted aP showed that TLR agonists alone induced better aP protection to a limited extent. A combination of several adjuvants was often required to stimulate strong antibody-based and cellular immune protection against each component of pertussis. The combination vaccines need to be used in early infancy, which introduces challenges regarding the safety of the novel adjuvanted pertussis–diphtheria–tetanus combination vaccines. Among the clinical trials on novel pertussis vaccines (excluding existing marketed aP vaccine manufacturing practices) (Table 2), the clinical trials on the application of pertussis vaccines combined with semisynthetic analog adjuvants (TQL1055) designed with Quillaja saponin (QS)-21 have been terminated. This resulted in a serious blow to the development of novel adjuvanted pertussis vaccines and indicated the difficulty in the development of novel adjuvanted pertussis vaccines. However, the pertussis vaccines with new adjuvants are used not only in combination with diphtheria–tetanus components but also as a new pertussis vaccine independent of the combined vaccines for booster immunization.

Currently, several attenuated pertussis vaccines are clinically available, including attenuated BPZE1 vaccines, which have been assessed through several clinical studies with ideal results. However, the BPZE1 vaccines need to be administered intranasally; therefore, they cannot be combined with existing diphtheria and tetanus vaccines to prepare a combined vaccine. Considering that the participants of the clinical studies on the existing BPZE1 vaccines comprise individuals aged 6–50 years (ClinicalTrials.gov Identifier: NCT03942406, NCT03541499, NCT05461131, NCT05116241, NCT02453048, and NCT01188512), these vaccines can be used as a booster immunization and for “cocoon strategy” to promote early Th2 responses induced by DTaP vaccination to shift to more protective Th1/Th17 and mucosal responses [84,124]. However, the suitability of live attenuated vaccines for neonatal vaccination still needs to be validated by preclinical and clinical studies considering that newborns’ immune systems differ from that of adults. In addition, although nucleic acid pertussis vaccines are less developed, the success of mRNA COVID-19 vaccines undoubtedly shows that they hold great promise [125]. In conclusion, there is still a long way to go for vaccine research on new pertussis. The current preclinical and clinical studies have indicated the direction and feasibility of future pertussis research.

## Figures and Tables

**Table 1:** Pertussis vaccination schedules across different countries for pregnant women.

Nation	Vaccination Schedule
Mauritius	1m/2m/3m/18m/5y/11y/pregnant
New Zealand/Niue	1m/3m/5m/4y/11y/pregnant
Burkina Faso	2m/3m/4m/pregnant
Belgium	2m/3m/4m/15m/5–6y/14–16y/pregnant
Netherlands	2m/3m/5m/11m/4y/pregnant
Israel	2m/4m/6m/12m/7y/13y/pregnant
Costa Rica	2m/4m/6m/15m/pregnant
El Salvador	2m/4m/6m/15–18m/4y/pregnant
Costa Rica	2m/4m/6m/15m/4–6y/14–16/pregnant
Brazil/The Bahamas	2m/4m/6m/15m/4y/pregnant
USA	2m/4m/6m/15m/4y/11y/pregnant
Uruguay	2m/4m/6m/15m/5y/11y/pregnant
Argentina	2m/4m/6m/15–18 m/6y/11y/pregnant
China Hongkong	2m/4m/6m/18m/6y/11y/pregnant
China Macao SAR	2m/4m/6m/18m/5–6y/7y/11y/pregnant
Colombia/Portugal	2m/4m/6m/18m/5y/pregnant
Dominican Republic/Guatemala/Honduras/Peru/Mexico/British Virgin Islands	2m/4m/6m/18m/4y/pregnant
Saudi Arabia	2m/4m/6m/18m/4–6y/pregnant
Panama	2m/4m/6m/18m/4–16y/pregnant
Australia	2m/4m/6m/4y/11–13y/pregnant
France	2m/4m/11m/6y/11–13y/25y/pregnant
Spain	2m/4m/11m/6y/pregnant
Romania	2m/4m/11m/6y/14y/pregnant
Bermuda	2m/4m/6m/4–6y/11–18y/pregnant
Ireland	2m/4m/6m/4y/12–13y/pregnant
Singapore	3m/4m/5m/18m/11y/pregnant
Slovenia	3m/5m/11–14m/9y/pregnant
Italy	3m/5m/11m/6y/12y/pregnant
Denmark	3m/5m/12m/5y/pregnant

The data was acquired from WHO [53]. m, month; y, year.

**Table 2:** Clinical trials involving new pertussis vaccines.

NCT Number	Status	Phase	Interventions	Inoculation Mode	S-Type
NCT04793620	Terminated	1	aP+TQL1055	I.M.	Adjuvant subunit
ACTRN12620001177943p	Completed	1	Tdap-CpG 1018	I.M.	Adjuvant subunit
NCT05116241	Recruiting	2	BPZE1	I.N.	Lived
NCT03942406	Completed	2	BPZE1	I.N.	Lived
NCT01188512	Completed	1	BPZE1	I.N.	Lived
NCT02453048	Completed	1	BPZE1	I.N.	Lived
NCT03541499	Completed	2	BPZE1	I.N.	Lived
NCT05461131	Recruiting	2	BPZE1	I.N.	Lived
NCT05136599	Not yet recruiting	1	Bordetella pertussis D420	I.N.	Lived
NCT03137927	Completed	1	GamLPV	I.N.	Lived
NCT04036526	/	1/2	GamLPV	I.N.	Lived
NCT05193734	Recruiting	2/3	aP+detoxied PT	I.N.	Recombined
NCT04102137	Completed	/	Pertagen (aP BioNet)	I.N.	Subunit, genetically inactivated (Arg9Lys and Glu129Gly)

I.M., intramuscular injection; I.N., intranasal immunization.

## Data Availability

Not applicable.
