# Correction: Nian et al. Coping Strategies for Pertussis Resurgence. *Vaccines* 2023, *11*, 889

**DOI:** 10.3390/vaccines12020151

**Published:** 2024-01-31

**Authors:** Xuanxuan Nian, Hongbo Liu, Mengyao Cai, Kai Duan, Xiaoming Yang

**Affiliations:** 1 National Engineering Technology Research Center for Combined Vaccines, Wuhan 430207, China; nianxuanxuan@126.com (X.N.);; 2 Wuhan Institute of Biological Products Co., Ltd., Wuhan 430207, China; 3 China National Biotech Group Company Limited, Beijing 100029, China

The authors would like to make the following corrections to this published paper [1].

In the fourth sentence of the Introduction, *Bordetella pertussis* was previously introduced to be non-motile; however, recent studies have shown that the bacterium pertussis has a flagellate structure, and all pertussis bacteria isolated from biological samples are motile. Thus, “non-motile” should be replaced with “motile”. A reference was added at the end of this sentence as reference 2 and the references thereafter were re-ordered:

2. Hoffman, C.L.; Gonyar, L.A.; Zacca, F.; Sisti, F.; Fernandez, J.; Wong, T.; Damron, F.H.; Hewlett, E.L. Bordetella pertussis can be motile and express flagellum-like structures*. mBio* **2019**, *10*, 1128. https://doi.org/10.1128/mbio.00787-19.

In the fifth sentence of Section 3, there was a typo: “DTaP vaccination …” should be changed to “Tdap vaccination …”.

In the first paragraph of Section 4.1, “SA” should be defined as “SA (a promoter, known as PTS1)”. It would have been more helpful to introduce the meaning of the abbreviation earlier in the text.

In the first paragraph of Section 4.1, the sentence “The use of the previously used pertussis vaccine, wP, has been almost eliminated because of more adverse reactions,” has been revised to “The use of the previously used pertussis vaccine, wP, has been largely replaced due to increased adverse reactions” for better understanding.

In the first paragraph of Section 4.3, “that can include Th2 and Th17 immune responses and stimulate humoral immune responses. However, the existing immunity” from the third and fourth sentences were deleted, and these two sentences were thus combined into one sentence “Licensed aP contains aluminum adjuvants may not be enough to prevent B. pertussis infection because of the lack of Th1 cellular immunity”.

In Section 4.4, we revised the sentence “Both the preclinical and clinical study of GamLPV provided the better immunogenicity and protective activity” into “Both the preclinical and clinical studies of GamLPV provided immunogenic activity.” The reason is that the word “better” may confuse readers because the immunogenicity and protective activity of GamLPV were not compared with any other vaccines, but between different doses. The next sentence, “BPZE1 was prepared by the knockout of the *B. pertussis* gene that encodes for skin necrosis toxin and replacement of the A mpG gene of pertussis by the A mpG gene of *Escherichia coli*, thus greatly reducing tracheal cytotoxin production”, was changed to “Attenuated *B. pertussis* is considered as a genetically engineered *B. pertussis* strain obtained by removing or altering genes involved in the production of three major *B. pertussis* toxins, PT, dermonecrotic toxin (DNT) and tracheal cytotoxin (TCT); this process greatly reduces tracheal cytotoxin production. BPZE1 expresses an enzymatically inactive PT by altering two key amino acids for the enzymatic activity of the toxin [108]”. This revision added the information that “inactive PTX was created by altering two key amino acids for the enzymatic activity of the toxin”. The following paper was cited in this new sentence as reference 108, and the references thereafter were re-ordered:

108. Mielcarek, N.; Debrie, A.S.; Mahieux, S.; Locht, C. Dose response of attenuated Bordetella pertussis BPZE1-induced protection in mice. *Clin. Vaccine Immun.* **2010,** *17*, 317–324.

In Table 2, the inoculation modes of the two clinical studies NCT05193734 and NCT04102137 have been corrected from “I.N.” to “I.M.” The corrected Table 2 is shown as below; moreover, clinical study NCT05136599 has been deleted because D420 is used for optimal and safe Bordetella pertussis doses.

The authors apologize for any inconvenience this may have caused and affirm that the scientific conclusions remain unaffected. The original publication has also been updated.

## Figures and Tables

**Table 2:** Clinical trials involving new pertussis vaccines.

NCT Number	Status	Phase	Interventions	Inoculation Mode	S-Type
NCT04793620	Terminated	1	aP + TQL1055	I.M.	Adjuvant subunit
ACTRN12620001177943p	Completed	1	Tdap-CpG 1018	I.M.	Adjuvant subunit
NCT05116241	Recruiting	2	BPZE1	I.N.	Lived
NCT03942406	Completed	2	BPZE1	I.N.	Lived
NCT01188512	Completed	1	BPZE1	I.N.	Lived
NCT02453048	Completed	1	BPZE1	I.N.	Lived
NCT03541499	Completed	2	BPZE1	I.N.	Lived
NCT05461131	Recruiting	2	BPZE1	I.N.	Lived
NCT03137927	Completed	1	GamLPV	I.N.	Lived
NCT04036526	/	1/2	GamLPV	I.N.	Lived
NCT05193734	Recruiting	2/3	aP + detoxied PT	I.M.	Recombined
NCT04102137	Completed	/	Pertagen (aP BioNet)	I.M.	Subunit, genetically inactivated (Arg9Lys and Glu129Gly)

I.M., intramuscular injection; I.N., intranasal immunization.
